# Mechanism of Action of Monoclonal Antibodies That Block the Activity of the Lethal Toxin of Bacillus Anthracis

**DOI:** 10.32607/actanaturae.11387

**Published:** 2021

**Authors:** Ya. O. Romanenko, A. K. Riabko, M. A. Marin, A. S. Kartseva, M. V. Silkina, I. G. Shemyakin, V. V. Firstova

**Affiliations:** Federal Budget Institution of Science State Research Center for Applied Microbiology and Biotechnology of Rospotrebnadzor, Obolensk, Moscow Region, 142279 Russia

**Keywords:** anthrax, monoclonal antibodies, toxin-neutralizing activity, cytometric analysis, protective antigen, lethal factor

## Abstract

Neutralization of the lethal toxin of Bacillus anthracis is an important topic of both fundamental medicine and practical health care, regarding the fight against highly dangerous infections. We have generated a neutralizing monoclonal antibody 1E10 against the lethal toxin of Bacillus anthracis and described the stages of receptor interaction between the protective antigen (PA) and the surface of eukaryotic cells, the formation of PA oligomers, assembly of the lethal toxin (LT), and its translocation by endocytosis into the eukaryotic cell, followed by the formation of a true pore and the release of LT into the cell cytosol. The antibody was shown to act selectively at the stage of interaction between Bacillus anthracis and the eukaryotic cell, and the mechanism of toxin-neutralizing activity of the 1E10 antibody was revealed. The interaction between the 1E10 monoclonal antibody and PA was found to lead to inhibition of the enzymatic activity of the lethal factor (LF), most likely due to a disruption of true pore formation by PA, which blocks the release of LF into the cytosol.

## INTRODUCTION

Anthrax is an anthropozoonotic infection caused by the gram-positive, aerobic, spore-forming, rod-shaped bacterium Bacillus anthracis. Depending on the route of bacterial administration, three primary forms of the disease are distinguished: gastrointestinal (alimentary route), cutaneous (contact route), and pulmonary (inhalation route). All forms of the disease can be fatal, but the airborne route of pathogen transmission is the most dangerous to human life [1, 2]. B. anthracis spores are from 1 to 5 µm in size, which enables them to easily enter the pulmonary alveoli upon inhalation. After penetration into the lungs, B. anthracis spores do not germinate but are quickly and efficiently phagocytized by alveolar macrophages and dendritic cells, which are then transported through the lymphatic ducts to the thoracic lymph nodes where the spores become vegetative cells that spread throughout the body and destroy cells [3].

The pathogenesis of anthrax is associated with two binary toxins and a capsule, which are encoded by the pX01 and pX02 plasmids. The pX01 plasmid encodes three components of the anthrax toxin: 83 kDa lethal factor (LF), 89 kDa edema factor (EF), and 85 kDa protective antigen (PA). The second plasmid, pX02, encodes the genes involved in the synthesis of the poly-D-glutamyl capsule. Removal of any plasmid decreases the virulence of bacteria [4].

The (effector) subunit A of anthrax binary toxins is represented by LF and EF, and the subunit B is represented by PA. Combining the A and B subunits results in the lethal toxin (LT), composed of PA and LF, and the edema toxin (ET), composed of PA and EF. The binary toxins were named according to their biological effects in animal models. Intradermal injection of ET (PA + EF) causes edema, and injection of a high concentration of LT (PA + LF) causes severe hypotension and death [5, 6].

**Fig. 1:**
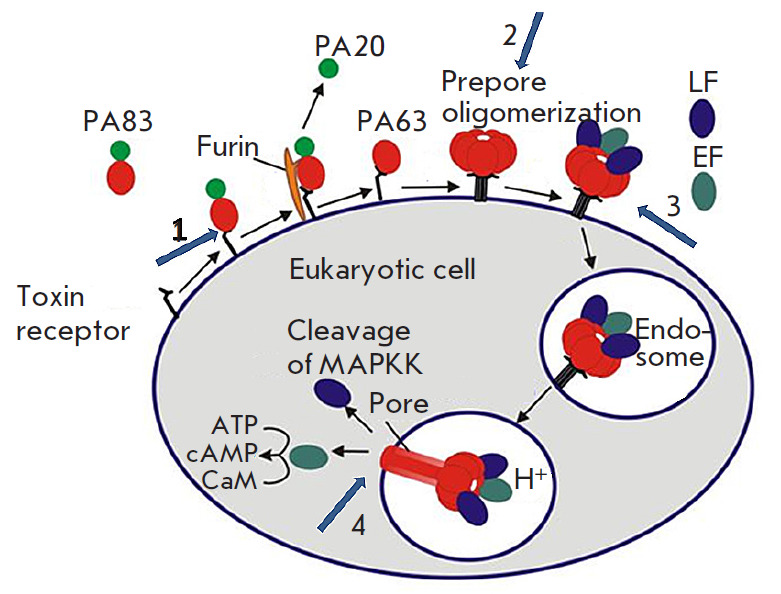
Schematic model of the assembly and activity of *B. anthracis *toxins. Numbered arrows indicate the key stages of the antitoxic activity of the 1E10 monoclonal antibody specifically interacting with domain IV of the *B. anthracis *protective antigen: 1. Binding of the mAb to the PA receptor; 2. Prevention of the assembly of an oligomeric PA63 prepore; 3. Inhibition of LF and EF binding to PA and prevention of endocytosis of toxin effector subunits; 4. Inhibition of conversion of the oligomeric PA63 prepore to the pore

The key subunit of toxins in the pathogenesis of anthrax is the PA that binds to receptors on the surface of immunocompetent cells and ensures the penetration of LF and EF into the cell. The receptor interaction of 83 kDa PA with the cell membrane is accompanied by the cleavage of a 20 kDa fragment by host furin-like proteases, resulting in the formation of 63 kDa PA. Monomeric PA63 oligomerizes and forms heptameric or octameric structures called prepores. Three LF or EF molecules bind to one heptamer, and 4 molecules bind to an octamer [7, 8]. After assembly of PA and LF/EF, the formed complex is internalized by the cell through clathrin-dependent endocytosis. The resulting endosome is gradually acidified. With changes in the environment’s pH level, PA changes its conformation, penetrates into the endosome, and forms a true pore for LF/EF translocation into the cytosol [9]. LF is a zinc metalloprotease that cleaves mitogen-activated protein kinase kinases (MAPKs) in the cytosol, which ultimately leads to cell apoptosis [10, 11]. Figure 1 presents all assembly stages and the toxic activity of LT and ET from B. anthracis as well as the key stages of the antitoxic activity of the monoclonal antibodies that specifically interact with the Bacillus anthracis protective antigen domain IV.

One of the interesting ways in which to protect the body from B. anthracis is to develop protective therapeutic antibodies. In recent years, therapeutic antibodies have become a potent tool in the fight against a whole range of pathologies [12, 13]. They are used as targeted agents for the elimination of pathological cells [14, 15]. Antibodies are very actively used as protective agents in toxic infections [11, 16, 17].

Figure 1 shows several possible pathways for disrupting the interaction between the toxin and the eukaryotic cell. In particular, it is possible to block the binding of PA to a cellular receptor or disrupt the formation of an adequate heptameric complex. It is also possible to block the binding of the toxin effector subunits to the prepore or inhibit the conversion of the prepore to the pore, which results in the inhibition of the kinase cascade.

To date, several LT-neutralizing monoclonal antibodies (mAbs) have been developed; most of these are murine mAbs, but there are also human toxin-neutralizing antibodies (Raxibacumab, GlaxoSmithKline). Nevertheless, the search for new, more effective LT-neutralizing antibodies continues [18].

Previously, we generated 1E10 mAb which exhibits specific activity against the PA domain IV [19]. The results of studies on the J774A.1 cell line and a mouse model showed a pronounced ability of 1E10 mAb to neutralize anthrax LT (some data are not published). The purpose of this study was to identify the inhibition mechanism of the LT cytotoxic effect by the monoclonal antibody 1E10.

## EXPERIMENTAL

In this study, we used recombinant proteins: protective antigen (rPA) according to [19] and lethal factor (rLF) according to [20]. The recombinant proteins have amino acid sequences of native B. anthracis PA and LF without signal peptides, as indicated in UniProtKB: P13423 (PAG_BACAN) and P15917 (LEF_ BACAN), respectively, fused with the N-terminal 6×His-tag and c-myc epitope. rPA and rLF expressed in E. coli BL21 (DE3) were purified from the cell lysate by chromatography using a cOmplete His-Tag

Purification Resin metal-chelate sorbent (Roche, Germany). Biotinylated recombinant proteins were prepared by conjugation to biotin sulfosuccinimidyl (sulfo-NHS) ester (Sigma, USA). Fluorophore-labeled recombinant proteins (rPA-FITC and rLF-Cy5) were obtained by conjugation to FITC (Thermo Fisher, USA) and the Cy5 mono-reactive dye (Amersham, UK).

**Evaluation of PA adhesion on the surface of J774A.1 macrophages in the presence of 1E10 mAb by flow cytofluorometry**

To assess the binding of PA to receptors on the surface of J774A.1 macrophages (ATCC®TIB-67™), 1 × 10^6^ cells per sample were incubated with fluorochrome-labeled rPA-FITC or rPA-FITC pre-incubated with 1E10 mAb at an equimolar ratio at 37°C for 1 h. J774A.1 cells were incubated with rPA-FITC or rPA-FITC + mAb at 37°C in a CO_2_ incubator with gentle stirring on an orbital shaker for 1 h. After incubation, all samples were washed three times with phosphate-buffer saline heated to 37°C (PBS; 137 mM NaCl, 2.7 mM KCl, 10 mM Na_2_HPO_4_, 1.76 mM KH_2_PO_4_, pH 7.4) and fixed with 1% formalin. The samples were analyzed on a FACSAria III flow cytometer (Becton Dickinson, USA) using the BD FACSDiva software (version 8.0). The cells were first analyzed using forward (FSC) and side (SSC) scatter gating to determine size and granularity, respectively. The ability of 1E10 mAb to inhibit adhesion of rPA-FITC on the cell surface was assessed by gating in SSC-A/FITC-A channels.

**Effect of 1E10 mAb on PA oligomerization**

Full-length 83 kDa rPA (PA83) was cleaved to produce PA63 and PA20. For that purpose, rPA was incubated with trypsin (Roche, Germany) at a concentration of 1 μg/mL at room temperature for 45 min. At the end of the incubation, the activity of the enzyme was inhibited by addition of a trypsin inhibitor from soybean (Roche, Germany) to a final concentration of 10 μg/mL. The sample was left under the same conditions. To stimulate oligomerization in the solution, all samples were added with rLF at a molar ratio of rPA : rLF = 2 : 1. At the next stage, 1E10 mAb was added to cleaved rPA at molar ratios of 1:1, 1:2, or 1:3. Control samples contained uncleaved rPA83, as well as PA63 + PA20, without addition of the antibody. All samples were incubated at 37°C for 60 min. Then, all samples were added with a 2-(N-morpholino) ethanesulfonic acid (MES) solution, pH 5.5, to a final concentration of 50 mM and incubated at 37°C for 30 min. For further separation in gradient (4–20%) PAGE under non-denaturing and non-reducing conditions, a sample loading buffer (according to Laemmli) without mercaptoetonol was added to the samples. After electrophoretic separation, the samples were transferred onto a Hybond-C Extra nitrocellulose membrane (GE Healthcare, UK) using an automatic Trans-Blot® Turbo™ Transfer System (Bio-Rad, USA). After transfer, the membrane was blocked by immersing it in skim milk with a fat mass fraction of no more than 0.5% and incubated on a thermostatted orbital shaker at 300 rpm and 37°C for 1 h. The membrane was washed with phosphate-buffer saline containing 0.05% Tween-20 (PBS-T; 137 mM NaCl, 2.7 mM KCl, 10 mM Na_2_HPO_4_, 1.76 mM KH_2_PO_4_, 0.05% Tween-20, pH 7.4). Then, the membrane was incubated with biotinylated anti-PA monoclonal mouse antibodies (clone 4F5 with specific activity against the PA domain III produced at the State Research Center for Applied Microbiology & Biotechnology) at a dilution of 5 μg/mL at 37°C for 1 h. After incubation, the membrane was washed three times with PBS-T, incubated with streptavidin conjugated to horseradish peroxidase (Streptavidin-Peroxidase Polymer, Ultrasensitive, Sigma, United States) at a dilution of 1 : 5,000, and washed six times with PBS-T. The reaction was visualized with a substrate mixture solution (0.05% diaminobenzidine (Sigma, USA), 0.015% H_2_O_2_ in PBS, pH 7.4). The reaction was stopped by washing with distilled water; then, the membrane was dried in air.

**Investigation of the effect of 1E10 mAb on LT endocytosis by flow cytometry**

To confirm the rPA–rLF interaction and subsequent LT endocytosis, we used flow cytometry. For this purpose, macrophages of the J774A.1 cell line (1 × 10^6^ cells per sample) were incubated with rPA-FITC and rLF-Cy5 in the presence or absence of 1E10 mAb. Solutions containing rPA-FITC and/or rLF-Cy5 in the presence or absence of mAb were pre-incubated at 37°C for 1 h and added to the cells. J774A.1 macrophages (1 × 10^6^ cells) separately incubated with rPA-FITC + rLF and rLF-Cy5 + rPA, as well as intact unstained J774A.1 cells, were used as controls. All samples with cells were incubated at 37°C in a CO_2_ incubator at gentle stirring on an orbital shaker for 30 min. After incubation, all the samples were washed three times with PBS heated to 37°C. Proteins were removed from the cell surface by adding a 0.01% trypsin solution, incubated at 37°C for 5 min, and washed three times with warm PBS. The cells were fixed with 1% formalin. Samples were analyzed on a FACSAria III flow cytometer. Gating was performed using forward (FSC) and side (SSC) scatter, and the effect of 1E10 mAb on the LF–PA interaction and LT endocytosis was assessed by gating in FITC-A and Cy5-A fluorescence channels.

**Effect of 1E10 mAb on specific LT activity**

Specific enzymatic activity of internalized LT was determined based on the presence of native or cleaved MEK. For that purpose, the following samples were prepared: J774A.1 mouse macrophages (1 × 10^7^ cells) were incubated in the presence of LT at a molar ratio of rPA : rLF = 5 : 1 with and without 1E10 mAb. LT was pre-incubated with or without mAbs at 37°C for 1 h; then, the solutions were added to the cells and incubated at 37°C in a CO_2_ incubator at gentle stirring on an orbital shaker for 30, 60, 120, and 240 min. J774A.1 macrophages (1 × 10^7^ cells) without addition of LT or mAbs were used as an intact control. After incubation, the cells were precipitated by centrifugation; the cell pellet was lysed in 0.5% Triton X-100; then, a sample loading buffer (according to Laemmli) with mercaptoetonol was added to the samples. The resulting samples were applied to gradient (4–20%) PAGE. After electrophoretic separation, the Western blot analysis was performed using the standard technique described above. The membrane was incubated with rabbit monoclonal antibodies to MEK1 + MEK2 (Abcam, UK, ab200179) at a dilution of 1 : 10,000. After incubation with specific monoclonal antibodies to MEK, the membrane was washed with PBS-T and incubated with a goat anti-rabbit IgG antibody, (H + L) HRP conjugate, (Merck, Germany) at a dilution of 1 : 1,000 in PBS. The interaction was visualized by a color reaction using diaminobenzidine as described above.

## RESULTS

**Evaluation of the ability of 1E10 mAb to inhibit rPA adhesion on the surface of J774A.1 macrophage-like cells**

**Fig. 2:**
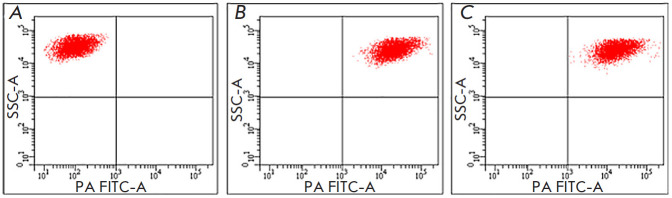
Effect of 1E10 antibodies on rPA adhesion on the surface of J774A.1 cells. (*A*) – cell samples incubated in medium in the absence of rPA-FITC and 1E10 mAb. (*B*) – cell samples incubated with rPA-FITC. (*C*) – cell samples incubated with rPA-FITC pretreated with 1E10 mAb

Figure 2 shows the distribution of J774A.1 cells incubated with medium (A), FITC-labeled PA (B), and FITC-labeled PA pretreated with 1E10 mAb (C). A comparative analysis of the presented cytograms indicates identity of the J774A.1 cell distributions in Fig. 2 B and C. In both cases, after incubation with rPA-FITC, pretreated with 1E10 mAb or not, an equally high level of cell fluorescence was observed, which was an indication of adhesion of rPA-FITC to their surface. These findings indicate that 1E10 mAb does not block the binding of rPA to the surface of eukaryotic cells.

**Evaluation of the ability of 1E10 mAb to block PA oligomerization**

**Fig. 3:**
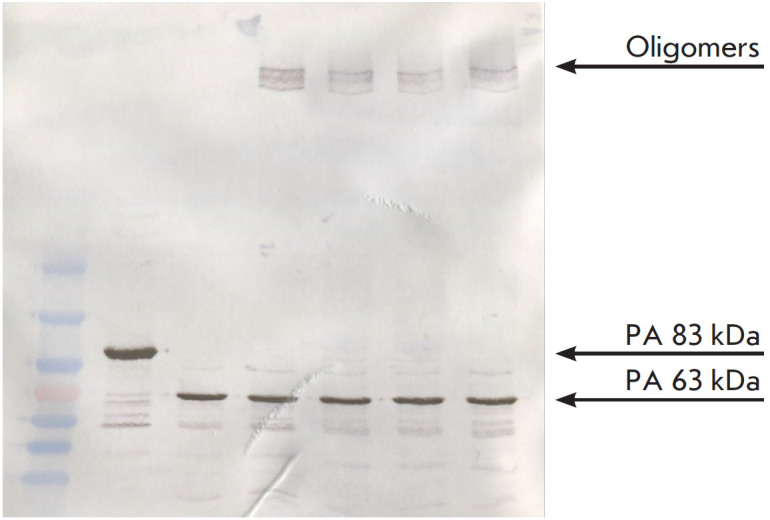
Assessment of the 1E10 mAb ability to block the formation of PA63 oligomers. *1 *– Molecular weight markers SM0671 (Fermentas, USA); *2 *– Control rPA (83 kDa); *3 *– PA63; *4 *– PA63 + rLF; *5 *– PA63 + 1E10 mAb (1:1) + rLF; *6 *– PA63 + 1E10 mAb (1:2) + rLF; *7 *– PA63 + 1E10 mAb (1:3) + rLF

The effect of 1E10 monoclonal antibodies on PA oligomerization was studied using Western blotting. The addition of 1E10 mAb to cleaved 63 kDa PA at antigen:mAb molar ratios of 1:1, 1:2, and 1:3 did not affect oligomer formation (Fig. 3). Therefore, 1E10 mAb does not prevent PA oligomerization and prepore formation.

**Effect of 1E10 mAb on the rLF–rPA interaction and LT endocytosis**

**Fig. 4:**
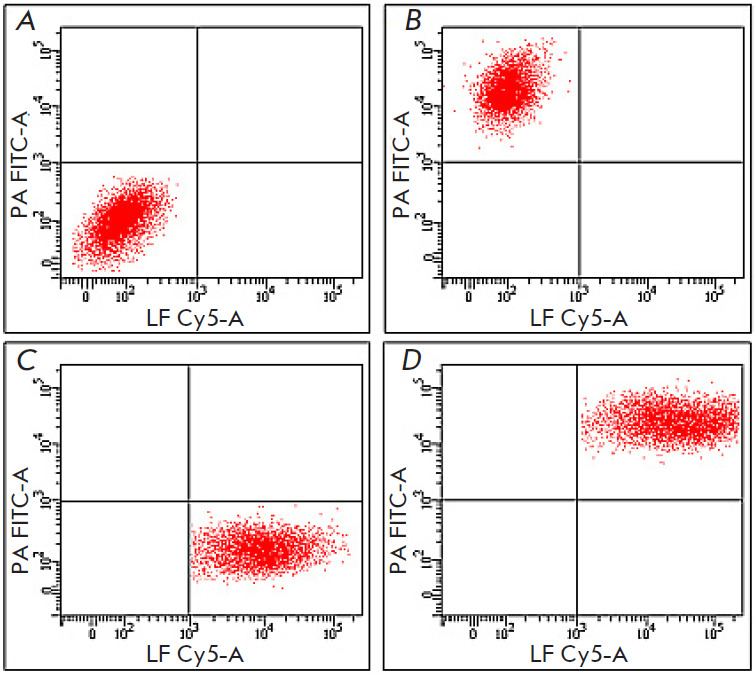
Assessment of the 1E10 mAb effect on the rLF–rPA interaction and LT endocytosis. (*A*) – cell samples incubated in medium without LT or mAb. (*B*) – cell samples incubated with rPA-FITC and unlabeled rLF. (*C*) – cell samples incubated with unlabeled PA and rLF-Cy5. (*D*) – cell samples incubated with 1E10 mAb-pretreated rPA-FITC and rLF-Cy5

Figure 4 shows cytograms of J774A.1 cells. The results of a cytometric analysis showed that all J774A.1 cells in the presence of fluorochrome-labeled rPA and rLF pretreated with 1E10 mAb were characterized by a high level of intracellular fluorescence of FITC and Cy5 dyes (Fig. 4D), which is an indication that 1E10 mAb is unable to block the rLF–rPA interaction and LT endocytosis.

**Effect of 1E10 mAb on specific LT activity**

**Fig. 5:**
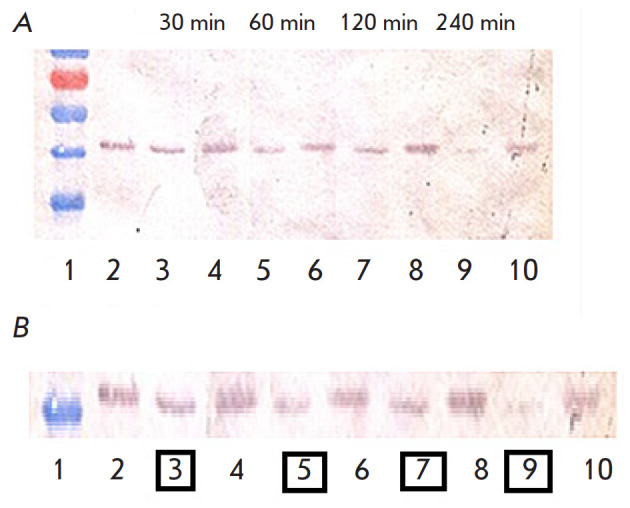
Effect of 1E10 mAb on the rLF enzymatic activity towards MEK1 and MEK2. (*A*) – Western blot results correlated with molecular weight markers. (*B*) – Enlarged image of Western blot results. *1 *– Molecular weight markers SM0671 (Fermentas, USA); *2 *– Control: intact J774A.1 cells; *3 *– J774A.1 cells + LT, incubation for 30 min; *4 *– J774A.1 cells + (LT + 1E10 mAb), incubation for 30 min; *5 *– J774A.1 cells + LT, incubation for 60 min; *6 *– J774A.1 cells + (LT + 1E10), incubation for 60 min; *7 *– J774A.1 cells + LT, incubation for 120 min; *8 *– J774A.1 cells + (LT + 1E10), incubation for 120 min; *9 *– J774A.1 cells + LT, incubation for 240 min; *10 *– J774A.1 cells + (LT + 1E10), incubation for 240 min

LF is a zinc-dependent endopeptidase that cleaves mitogen-activated protein kinases (MAPKKs), in particular MEK1 and MEK2, with removal of a 1.2 kDa peptide. Figures 5 A and 5B (lanes 3, 5, 7, 9) show that LT causes cleavage of MEK1 and MEK2 d, while LT pretreated with the 1E10 monoclonal antibody leaves MEK1 and MEK 2 intact. During long-term incubation (240 min), samples prepared from cell culture incubated with LT without addition of the mAb contained lower amounts of MEK1 and MEK 2 (Fig. 5 A and B, lane 9), which probably indicates the passage of rLF through the pore into the cell cytosol and the enzymatic activity of rLF towards MEK1 and MEK2, leading to cell apoptosis. Therefore, we have found that the 1E10 mAb–rPA interaction inhibits the enzymatic activity of LT towards MEK1 and MEK2.

## DISCUSSION

In the Russian Federation, treatment of anthrax involves antibiotics and equine anti-anthrax immunoglobulin (33^rd^ Central Research Institute of the Ministry of Defense of the Russian Federation, Russia) that contains polyclonal antibodies to antigens of the B. anthracis STI-1 vaccine strain and anthrax toxins. In generalized anthrax, antibiotics are not effective and the equine anti-anthrax immunoglobulin can cause side effects, including anaphylactic shock and serum sickness [20, 21]. The use of monoclonal antibodies provides a predictable efficacy in neutralizing the anthrax toxin, and the use of chimeric antibodies reduces allergization of the body. The use of mAbs against PA is the most promising strategy for the treatment of anthrax, which provides inhibition of the toxic effect of anthrax toxins. This is due to the fact that PA is an essential LT subunit responsible for the toxic activity, which enables penetration of LF and EF into the cell cytosol. Our previously developed 1E10 mAb to PA domain IV had exhibited its lethal toxin-neutralizing activity and is considered a basis for the development of chimeric therapeutic mAbs. In this work, we analyzed the stages that might be affected by the lethal toxin-neutralizing activity of 1E10 mAb.

An analysis of the interaction of rPA with the surface membrane of J774A.1 macrophage-like cells, rPA oligomerization with prepore formation, rLF–rPA interaction, and LT endocytosis in the presence of 1E10 mAb revealed a lack of inhibitory activity 1E10 mAb towards these processes.

We supposed that 1E10 mAb, binding to PA, might disrupt the conformational rearrangements of PA during the formation of the pore for LF penetration into the cytosol, where it becomes enzymatically active. LF is known to hydrolyze MEK1 and MEK2 in the N-terminal region, with the cleavage of a 1.2 kDa peptide. MEK1 and MEK2 are mitogen-activated protein kinases (MAPKKs) that are involved in a variety of cellular processes. Using the MEK1 + MEK2 specific antibody, we showed that opsonization of PA by the 1E10 monoclonal antibody leads to the inhibition of the LT enzymatic activity towards MEK1 and MEK2.

## CONCLUSION

Therefore, the mechanism of LT inhibition by the 1E10 monoclonal antibody involves the inhibition of the enzymatic activity of LF towards MEK1 and MEK2, which is likely associated with a disruption of the pore formation process and the impossibility of LF release into the cytosol.

In our opinion, this study has clearly demonstrated the potential of using therapeutic antibodies in the fight against infections. It should be noted that the COVID 19 pandemic clearly reinforced this conclusion. Along with great success in the development of vaccines, the use of virus-neutralizing antibodies in certain categories of patients is considered appropriate [22]. The generation of individual patient B-cell clones producing neutralizing antibodies is based on recently developed microfluidic technologies [23, 24]. These technologies have enabled a real breakthrough in the development of SARS-CoV-2-neutralizing therapeutic antibodies [25, 26]. The specificity of antibodies against the lethal toxin of Bacillus anthracis, which were produced in this study, may be further modified using combinatorial biology methods.

## Abbreviations

B. anthracis: Bacillus anthracis
LT: lethal toxin
LF: lethal factor
rLF: recombinant lethal factor of B. anthracis
EF: edema factor
PA: protective antigen
rPA: recombinant protective antigen of B. anthracis
rPA-FITC: recombinant protective antigen of B. anthracis conjugated to fluorescein- 5-isothiocyanate
rLF-Cy5: recombinant lethal factor of B. anthracis conjugated to a fluorescent dye Cy5
MAPKK: mitogen-activated protein kinase kinase
MEK: mitogen activated kinase
mAb: monoclonal antibody
TNA: toxin-neutralizing activity
PBS: phosphate-buffer saline
MES: 2-(N-morpholino) ethanesulfonic acid
PAGE: polyacrylamide gel
PBS-T: phosphate-buffered saline with 0.05% Tween-20.
